# The effects of daylight exposure on melatonin levels, *Kiss1* expression, and melanoma formation in mice

**DOI:** 10.3325/cmj.2020.61.55

**Published:** 2020-02

**Authors:** Percin Pazarci, Halil Kaplan, Davut Alptekin, Mehmet Yilmaz, Umit Lüleyap, Ergin Singirik, Aykut Pelit, Halil Kasap, Arash Yegani

**Affiliations:** 1 Department of Medical Biology, Çukurova University Faculty of Medicine, Adana, Turkey; 2 Department of Pharmacology, Çukurova University Faculty of Medicine, Adana, Turkey; 3 Department of Biophysics, Çukurova University Faculty of Medicine, Adana, Turkey; 4 Department of Pharmacology and Toxicology, Hatay Mustafa Kemal University Faculty of Veterinary Medicine, Antakya, Turkey; Pazarci et al: Effects of daylight exposure on melatonin, *Kiss1* expression, and melanoma in mice

## Abstract

**Aim:**

To determine how daylight exposure in mice affects melatonin protein expression in blood and *Kiss1* gene expression in the hypothalamus. The second aim was to assess the relationship between skin cancer formation, daylight exposure, melatonin blood level, and kisspeptin gene expression level.

**Methods:**

New-born mice (n = 96) were assigned into the blind group or daylight group. The blind group was raised in the dark and the daylight group was raised under 12 hours light/12 hours dark cycle for 17 weeks. At the end of the 11th week, melanoma cell line was inoculated to mice, and tumor growth was observed for 6 weeks. At the end of the experiment, melatonin level was measured from blood serum and *Kiss1* expression from the hypothalamus.

**Results:**

The blind group had significantly higher melatonin and lower *Kiss1* expression levels than the daylight group. Tumor volume was inversely proportional to melatonin levels and directly proportional to *Kiss1* expression levels. Tumor growth speed was lower in the blind than in the daylight group.

**Conclusion:**

Melatonin and *Kiss1* were shown to be involved in tumor suppression*.* They were affected by daylight and were mutually affected by each other.

Melatonin is an endocrine hormone produced by the pineal gland and several body tissues, and its blood levels are inversely proportional to the amount of light received throughout the day (1,2). Melatonin alterations regulate the circadian rhythm of many bodily functions (3). It has been shown that circadian rhythm disruptions may lead to impaired thyroid-stimulating hormone (TSH) secretion, increase in nocturnal cortisol secretion, changes in lipid and glucose metabolism, changes in cytokine balance, and inhibition of antioxidant genes (4). Melatonin also regulates the production of kisspeptin, a protein coded by *Kiss1* and synthesized mostly in the hypothalamic tissue (5). It has been shown that kisspeptin levels vary depending on melatonin blood concentration (6).

Melatonin’s tumor suppressor properties are the subject of considerable research. Its antioxidant properties and DNA protective features (nuclear and mitochondrial) have been extensively confirmed (7,8), while cell culture and animal studies have emphasized its role in the suppression of different tumor types (9,10). For example, the incidences of breast cancer, stomach cancer, and skin cancer were lower in blind people, whose melatonin levels were consistently higher than those in sighted individuals (11,12).

Melatonin and kisspeptin synthesis are both affected by daylight exposure (13). Also, decreased melatonin blood levels lead to increased kisspeptin synthesis in the hypothalamus (14,15). Although kisspeptin’s primary function is the seasonal control of reproduction, various studies also showed its antimetastatic role (16,17).

The relationships between daylight exposure and melatonin, daylight exposure and kisspeptin, and kisspeptin and melatonin have been widely investigated, but there have been no detailed studies on their mutual effects. This study aimed to determine how daylight exposure in mice affected melatonin blood levels and the rate of kisspeptin synthesis in the hypothalamus. In addition, we investigated the relationship between skin cancer formation, daylight intake, melatonin blood level, and kisspeptin synthesis rate.

## Material and methods

The study, conducted in 2017, used 96 newborn BALB/c albino mice obtained from the Çukurova University Faculty of Medicine Experimental Medicine Research and Application Center. No inclusion or exclusion criteria other than age and sex were applied. This study was approved by the Ethics Committee of the Çukurova University Faculty of Medicine Experimental Medicine Research and Application Centre.

## Mice groups and experimental workflow

The mice were assigned to the blind group (n = 48) or the daylight group (n = 48). Each group was further divided into the control (n = 12) and melanoma (n = 36) subgroups. All subgroups had an equal number of male and female mice. The blind group was housed with their mothers in a dark room (0 lux) one week after birth. Since visual skills in mice develop 10-14 days after birth, the exposure to darkness was used to imitate blindness from birth (18). The daylight group was housed with their mothers in a room with normal daylight (4000 lux, 12 hours daylight, 12 hours dark) one week after birth. All mice were separated from their mothers at the end of week 3 and were raised under appropriate conditions (unlimited Laboratory Diet 5K52, unlimited water, 20°C, 50% humidity). At the end of week 11, the mice in the melanoma subgroups were subcutaneously injected with B16F10 cell line and raised for 6 more weeks (17-week old mice). The tumor sizes were measured weekly with a caliper. At the end of week 17, tumor sizes were measured, blood samples were taken, and the hypothalamuses were removed.

### Melanoma cell line injection

The cell lines were prepared and injected according to the modified protocol by Overwijk and Restifo (19). B16F10 cells, which were in the active dividing state in the cell culture, were collected and diluted with DMEM to a concentration of 10^6^ cells/mL. Melanoma cell solution of 100 μL (10^5^ cells) was administered subcutaneously to the abdominal areas.

### Tumor size measurement and volume calculation

The measurements were made between the longest transverse (width) and the longest longitudinal (length) sections. The short section was considered to be the tumor width and the long section was considered to be the tumor length. Tumor volume was calculated by the formula: tumor volume = width × width × length/2 (20,21).

### Determination of melatonin concentration

Melatonin blood concentration was determined with ELISA kit (SunredBio Inc., Shanghai, China, detection range: 15.6-1000 pg/mL) according to the manufacturer’s protocol. Since the melatonin level was measured from the blood serum, no standardization was done.

### Determination of Kiss1 expression

*Kiss1* expression was determined in the hypothalami. The expression level was determined with real time quantitative polymerase chain reaction by using the TaqMan Gene Expression Assay (ThermoFisher Scientific Inc, Waltham, MA, USA) containing the FAM stained probe designed for *Kiss1* gene. RNA was isolated with TRIzol method (22). Complementary DNA was synthesized with High-Capacity cDNA Reverse Transcription Kit (Applied Biosystems Inc., Foster City, CA, USA). The expression level of *Kiss1* gene was determined with ΔCt method using β-actin gene as reference (23). One of the samples was accepted as “1” and the expression levels of other samples were determined relatively.

### Statistical analysis

Normality testing was conducted with the Kolmogorov-Smirnov test. Significance of differences between the groups in melatonin and *Kiss1* levels was assessed with the independent *t* test, while the significance of differences between the groups in the rate of tumor volume change was assessed with the two-way ANOVA. Correlations between melatonin and *Kiss1* values and tumor volumes were assessed with the Pearson correlation analysis. The level of statistical significance was set to 0.05. The analysis was conducted with Graphpad Prism 6 software (GraphPad Software Inc, San Diego, CA, USA).

## Results

At the end of the experiment, 87 mice survived. Nine mice (1 healthy from the daylight group and 8 injected mice, 5 from daylight and 3 from blind group) died from unknown causes and were excluded from the study.

The blind group had significantly higher melatonin level (17.26 ± 0.97 ng/L vs 12.77 ± 0.53 ng/L *P* ≤ 0.001, *t* = 3.980) and significantly lower *Kiss1* level than the daylight group (5.89 ± 1.21 vs 13.00 ± 2.92 *P* = 0.024, *t* = 2.306 for *Kiss1*).

Healthy mice had significantly higher melatonin level (16.09 ± 1.26 ng/L vs 9.59 ± 0.98 ng/L, *P* = 0.002, *t* = 3.440) and significantly lower *Kiss1* expression than tumor-bearing mice (3.08 ± 1.15 vs 11.96 ± 3.07, *P* = 0.003, *t* = 3.280).

**Figure 1:**
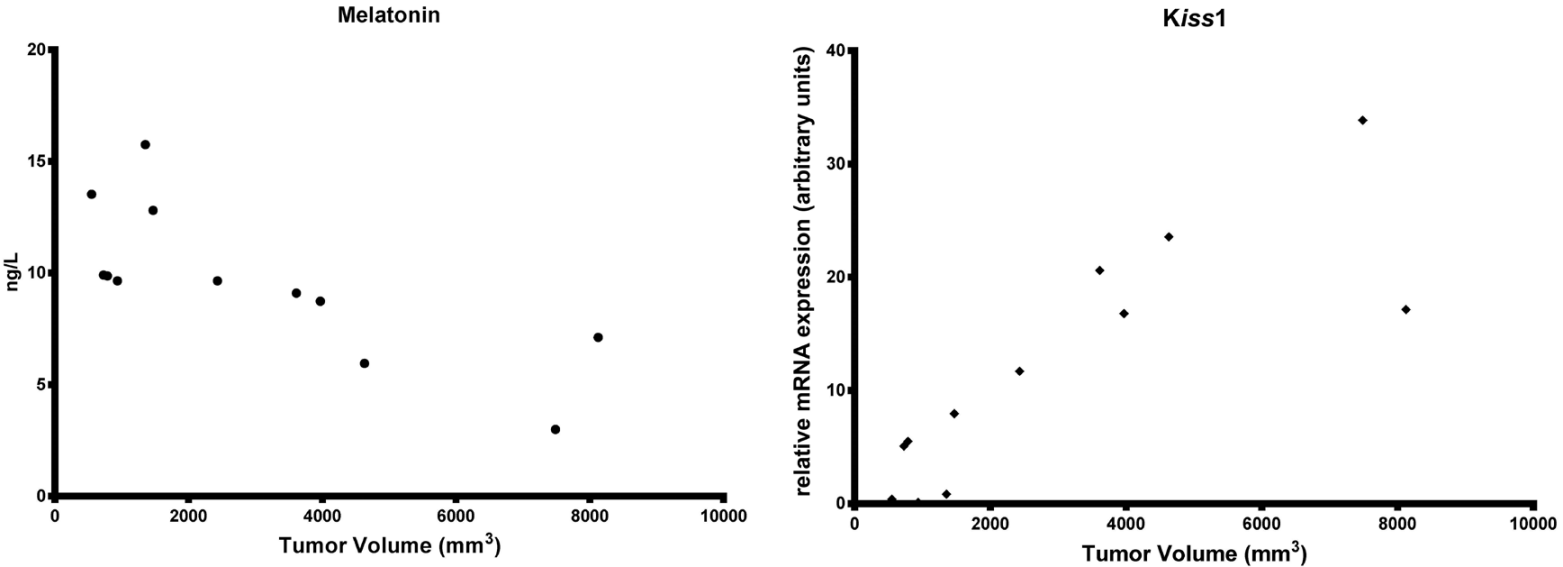
The relationship between melatonin and *Kiss1* and tumor volumes at the end of the experiment (*P* < 0.05).

A tumor was formed in 12 of 72 mice injected with a melanoma cell line. Six of these were female and 6 were in the daylight group. There was no difference between the groups and sexes in the number of tumor-bearing mice. The weekly change of tumor volume from the injection to sacrifice is shown in Table 1. There was a strong inverse correlation (correlation coefficient = -0.766, *P* = 0.004) between melatonin levels and tumor volumes and a strong positive correlation (correlation coefficient = 0.849, *P* = 0.001) between *Kiss1* expression and tumor volumes (Figure 1).

**Table 1:** Tumor volumes (mm^3^) of melanoma bearing mice by week

Mouse*	Week 1^†^	Week 2	Week 3	Week 4	Week 5	Week 6
**Blind group**						
**F**	-	18.00	87.50	486.00	936.00	936.00
**F**	-	32.00	320.00	2560.00	7488.00	7488.00
**F**	-	22.50	245.00	1764.00	4630.50	4630.50
**F**	-	6.00	56.00	288.00	726.00	726.00
**M**	-	13.50	56.00	320.00	786.50	786.50
**M**	-	6.00	31.50	220.50	550.00	550.00
**Daylight group**						
**F**	-	32.00	486.00	3240.00	3971.00	3971.00
**F**	-	40.00	936.00	6083.50	8125.00	8125.00
**M**	-	18.00	650.00	2601.00	3610.00	3610.00
**M**	-	13.50	550.00	1912.50	2432.00	2432.00
**M**	-	13.50	288.00	936.00	1470.00	1470.00
**M**	-	6.00	220.50	786.50	1352.00	1352.00

*F – female; M – male.

†Week numbers represent weeks after injection.

**Figure 2:**
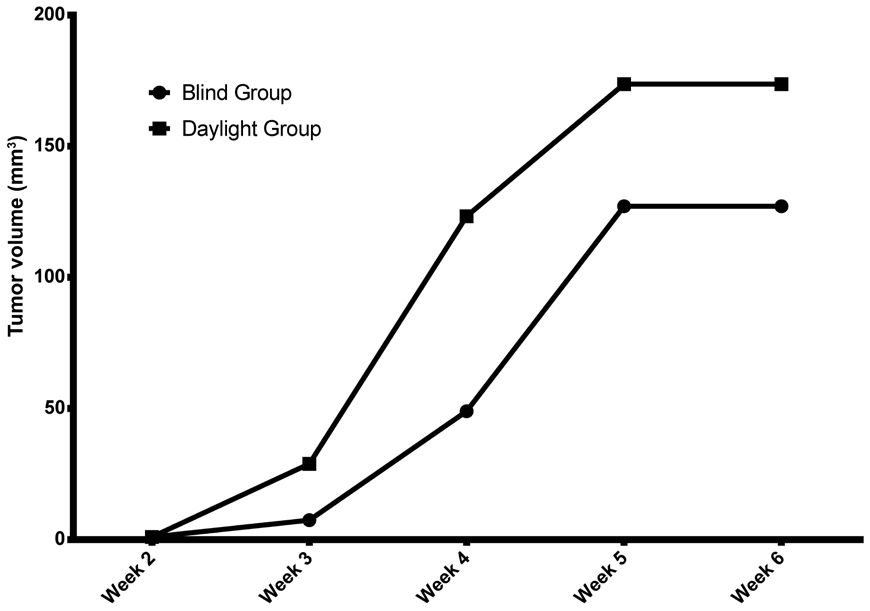
Tumor volume rates in the daylight and blind group (*P* < 0.05).

Tumor volumes measured each week (Table 1) were divided by the values at the week 2, when tumors were first spotted, and the growth rate was determined for every week after tumor formation (Table 2). The tumor volumes in the daylight group grew significantly faster than those in the blind group (*P* = 0.026) (Figure 2).

**Table 2:** Changes in the rate of tumor growth by week

Mouse*	Week 2^†^	Week 3	Week 4	Week 5	Week 6
**Blind group**					
**F**	1.00	4.86	27.00	52.00	52.00
**F**	1.00	10.00	80.00	234.00	234.00
**F**	1.00	10.89	78.40	205.80	205.80
**F**	1.00	9.33	48.00	121.00	121.00
**M**	1.00	4.15	23.70	58.26	58.26
**M**	1.00	5.25	36.75	91.67	91.67
**Daylight group**					
**F**	1.00	15.19	101.25	124.09	124.09
**F**	1.00	23.40	152.09	203.13	203.13
**M**	1.00	36.11	144.50	200.56	200.56
**M**	1.00	40.74	141.67	180.15	180.15
**M**	1.00	21.33	69.33	108.89	108.89
**M**	1.00	36.75	131.08	225.33	225.33

*F – female; M – male.

†Week numbers represent weeks after injection.

## Discussion

In this study, mice kept in darkness (blind group) had a slower tumor growth rate in comparison with mice exposed to daylight conditions (daylight group). Furthermore, the blind group had significantly higher melatonin level and significantly lower *Kiss1* level than the daylight group. One of the most important factors that regulate the melatonin cycle is the light stimulation of the retinal nerves (24,25). Individuals with partial visual impairment who could perceive light had slightly deviated melatonin cycle, whereas individuals with complete visual impairment, not able to perceive light, had an abnormal cycle during the day (26). In addition, individuals who had lost both eyes had disrupted circadian rhythm and a spontaneous melatonin cycle (27). The higher melatonin levels in the blind group observed in this study could be attributed to the irregular melatonin cycle in the blind, leading to higher melatonin levels during the day (26,27). Both groups were sacrificed during the daytime to detect the baseline blood melatonin levels.

The *Kiss1* level was significantly higher in the daylight than in the blind group. Kisspeptin synthesis is directly proportional to the duration of daylight exposure (28), because kisspeptin controls reproductive behavior, which is increased in the long-day season (29).

Melatonin levels were very low in tumor-bearing mice compared with healthy mice. Grinevich and Labunetz (30) also found very low melatonin levels in melanoma patients compared with healthy individuals. Low melatonin levels in tumor-bearing mice may be related to the circadian rhythm disruption. Another possibility is that mice with lower melatonin levels developed melanoma, while mice with higher melatonin levels were able to protect themselves from tumor formation. However, despite the different melatonin levels, there was no difference between the daylight and blind group in the number of tumor-bearing mice, which makes this possibility less probable. *Kiss1* level was much higher in tumor-bearing than in healthy mice. If we take into account kisspeptin’s antimetastatic and anticancer properties, it can be concluded that the hypothalamic synthesis of kisspeptin was increased because of tumor formation. Contrary to our findings, Shirasaki et al (31) reported that *Kiss1* expression was reduced in metastatic melanomas. This difference can be explained by the fact that our mice did not have metastases. In addition, tumor volume strongly inversely correlated with melatonin, whereas it strongly directly correlated with *Kiss1*. Tumor volumes increased as the melatonin level decreased, which indicates the protective effect of melatonin on melanoma formation. Tumor volumes also increased with the increase in *Kiss1* expression level, and considering the fact that the mice had no metastases, this observation may be explained by the potential effect of changed kisspeptin synthesis on metastasis inhibition. However, this interpretation has to be confirmed by kisspeptin assessment in tumor tissues. In addition, the study did not analyze both protein and gene expression of melatonin and kisspeptin – we analyzed melatonin protein expression in blood and kisspeptin gene expression level in the hypothalamic tissue. 

The main limitation of the study was the fast melanoma growth. Fast growing tumors became life threatening for mice, preventing us from extending the study observation period to observe metastasis development, which was one of the original study aims.

Our results showed that melatonin and *Kiss1* were important tumor suppressors and were highly affected by daylight. In addition, these two tumor suppressors were mutually affected by each other. Future studies should analyze both protein and gene expression of melatonin and kisspeptin in tumor tissues to answer the remaining questions, particularly how to generate more slowly progressing cancers in mice.
